# Relation of Animal Diseases to the Public Health

**Published:** 1885-01

**Authors:** 


Relation of Animal Diseases to the Public Health.—Apropos of the subject of the preceding article we find in Dr. Billings' book with the above title some statements which will illustrate the point.

Referring to the fact that in the thirty years during which the English ports were thrown open to the introduction of foreign cattle, the loss has been 5,500,000 head of cattle valued at £83,000,000, caused by pleuro-pneumonia and "foot and mouth disease," and in view of the possibility of an epidemic in this country, the Doctor says, "there is but one rule for its treatment: *No temporizing*. Immediate slaughter and redemption by the government."

"It is highly probable that were the real facts known, the ravages of contagious diseases have cost the people more in the last hundred years of our existence than our national debt amounts to."

"Every one should know that, if not absolutely preventable, yet it is possible for a competent Veterinary Police to reduce these losses to a very low minimum." "No prevention can be hoped for until we have a national code of police laws and an efficient body of educated veterinarians to execute them."

What can we expect from the present condition of legislation in this matter, when we hear a prominent Boston merchant exclaim on learning that there was no law to prevent his sending trichinous pork to market, "Thank God! there is some freedom left in Massachusetts."

Veterinarians will find in Dr. Billings' book much important information and many practical hints, which will be highly valuable to them in studying the actual condition of the cattle industry, the dangers to which it is subjected, and the protection which it needs. C.

